# Stakeholder perspectives and the challenges in implementing hepatitis C elimination policy in Pakistan

**DOI:** 10.3389/fpubh.2025.1540689

**Published:** 2025-07-16

**Authors:** Naeem Asim, Wenbiao Hu, Liang Qiao, Usman Ali Khan, Zaka Un Nisa

**Affiliations:** ^1^Islamia University of Bahawalpur, Bahawalpur, Pakistan; ^2^Queensland University of Technology, Brisbane, QLD, Australia; ^3^The University of Sydney, Darlington, NSW, Australia; ^4^Primary and Secondary Healthcare Department, Lahore, Punjab, Pakistan; ^5^Quaid-i-Azam University, Islamabad, Pakistan

**Keywords:** HCV, hepatitis, Pakistan, policy, elimination, control

## Abstract

Despite significant achievements in combating hepatitis C (HCV), HCV remains a major global public health issue with approximately 71 million cases and 400,000 deaths annually. The international community has taken several important steps for HCV control, including the Global Health Sector Strategy (2016–2021), the Global Hepatitis Action Plan (2011), and the United Nations Sustainable Development Goals (UN-SDGs) for 2030. However, achieving the World Health Assembly’s goal of eliminating HCV by 2030 treating 90% of all infected individuals remains a significant challenge, particularly in Pakistan. This study delves into the perspectives of key stakeholders involved in the hepatitis elimination policy and its implementation in Pakistan, identifying barriers to effective policy execution and highlighting motivating factors. Through a phenomenological approach, interviews were conducted with ten key informants, including policymakers, clinicians and provincial hepatitis program personnel. Thematic analysis uncovered several critical themes: perceptions of hepatitis and elimination policies, the feasibility of achieving elimination goals, international collaborations, policy development, gaps in epidemiological data, and the influence of political contexts. The findings emphasize the need for a robust national database, stronger political commitment, better resource allocation, and a more integrated healthcare system. Drawing on successful models, such as Egypt, the study suggests that Pakistan must adopt comprehensive and coordinated strategies to meet the WHO targets and eliminate hepatitis by 2030.

## 1 Introduction

The triumphs in the battle against hepatitis over the past several years are well documented. Despite these advancements, hepatitis C remains a leading cause of mortality and morbidity worldwide, with approximately 71 million cases of hepatitis C and 400,000 deaths every year (1). In the past several years, score of solid steps have been taken at world level, including the Global Health Sector Strategy (2016–2021) (2), Vaccination programs, mass awareness campaigns, expansion & enhancement of testing and screening initiatives, betterment in access to treatment (3), Global Hepatitis Action Plan in 2011 (4) and United Nations Sustainable Development Goals (UN-SDGs) for 2030 (5). Across the globe, merely 12 countries are expected to eliminate hepatitis C by 2030, with Italy, the United Kingdom and Spain among the prominent nations (6, 7). Egypt has commendably transformed itself from one of the highest burdens of HCV in the world to the lowest (10–0.38%) in the last one decade, achieving the “gold tier” status vis-a-vis eliminating hepatitis C as per criteria of WHO (8, 9).

In 2016, the World Health Assembly committed to eliminating hepatitis C by 2030 through the implementation of a combination of high-impact interventions, that are projected to end hepatitis with the treatment of 90% of all people with HCV (7). The WHO has urged all countries to invest in eliminating hepatitis through sustained financial support to the eradicating services within their universal health coverage plans (10). Meeting held in Amsterdam on 17–18 November 2017 brought together virologists, public health professionals, and clinicians to discuss the challenges and major gaps in eliminating HCV (11). In the cascade of care optimization, stigmatization and lack of high-quality epidemiological data were identified as the main challenges in the elimination of HCV (11). The achievement of this goal, apart from an effective treatment strategy, requires an adequate financial structure, policies to address the new infection prevention, stakeholder involvement, and political will (10). According to a study approximately $58.7 billion is required to eliminate hepatitis C in low-middle-income countries (LMICs), comprising 53 million people with HCV infection (12). A $6 billion investment every year would help prevent 4.5 million premature deaths by 2030 and more than 27 million deaths beyond the set date (10, 13).

Globally, Pakistan ranks second in terms of HCV infection rate, with almost one in every 20 people infected with HCV. HCV infection incurs a mortality of approximately 366,000 every year (14–17). The epidemic is of huge scale and highly prevalent across all the provinces, with the highest prevalence reported in Punjab (around 9%) and Sindh (approximately 5.6%) (18, 19). HCV prevalence has been mounting over the past several years, indicating ongoing rapid transmission due to persistent risk factors like unsafe large number of injections, unscreened or poorly screened blood transfusions, and poor sterilization practices, unsafe surgeries, unhygienic barbers and beauty salon practices, injection drug users and unsafe dental procedures. Injection drug use and healthcare exposures drive new infections. Without stronger interventions, cases could exceed 11 million by 2035, causing high rates of liver disease and over 130,000 deaths annually (20).

Genotype 3a is the most common genotype accounting for 63% of the cases (21). Currently, the government’s funds for hepatitis treatment and control are insufficient to yield the expected results. To reach the WHO set target of hepatitis C elimination, Pakistan needs to screen an average of 18.9 million people, 1.1 million treatments and prevention of 470,000 new infections every year (22). Some of the factors contributing to the inadequate control of HCV infections in Pakistan include lack of research, underreporting, lack of reliable epidemiological data, lack of awareness, fragile health system, lack of sustained funding, negligence, corruption and weak political will (16, 22).

Although there are few studies directly examining the attitudes of health providers toward implementing hepatitis prevention policies, the broader contest offers valuable insight into the global fight against hepatitis and its elimination in Pakistan. Understanding the stakeholders’ attitudes, knowledge, perceptions and involvement in both facilitating and overcoming barriers to policy implementation in Ghanaian healthcare facilities through Lipsky’s theory of street-level bureaucracy revealed that frontline workers often make decisions based on their professional judgment, adjusting policies according to costs, resources and practical considerations (23). This highlights that the attitudes of stakeholders are a key factor influencing health policy implementation (23). Additional factors impacting implementation include adequate of human resource, lack of involvement of local implementors, poor leadership, inadequate human resources, a fragile healthcare system, and management deficiencies (24). This study aimed to investigate the stakeholders (hepatitis policy makers, clinicians and provincial hepatitis program personnel) understanding of the hepatitis prevention program in Pakistan. Their perspectives on hepatitis elimination are of the utmost significance, as they possess firsthand knowledge of all the issues Pakistan faces in the elimination efforts of hepatitis.

## 2 Methods

A total of 10 key informants (thereby referred to as “participants”) were purposefully selected for their specific interest and involvement in hepatitis elimination policy implementation in Pakistan. These individuals include participants from the provincial hepatitis programs in all four endemic provinces (Punjab, Khyber-Pakhtunkhwa, Sindh and Baluchistan). Among them were the policymakers, clinicians and provincial hepatitis control program personnel. In the provinces, program managers serve as gatekeepers of policy implementation. Therefore, including these participants was deemed highly pertinent. Previously, no study has been carried out considering the perspectives of all stakeholders involved in policy-making and implementation.

Interviews with 2–10 participants are generally sufficient to achieve saturation in phenomenological studies (25, 26). The participants were identified through the researcher’s network of contacts within the hepatitis community. All participants in this study expressed their willingness to be interviewed. The interviews were tape-recorded after obtaining informed consent from the participants and assuring them that all information would remain confidential and their details would not be shared. Five interviews were conducted face-to-face, and five were carried out through Zoom, owing to funding and distance issues. Each interview lasted about 40–60 min. The inquiries pertained to understanding hepatitis prevention policy and its feasibility, encompassing the factors aiding or hindering effective implementation. A professional transcriber expertly transcribed the interviews verbatim. The authors meticulously reviewed all transcriptions while listening to the audio recordings to verify accuracy. Subsequently, all transcriptions were repeatedly reviewed to identify emerging and significant themes. Coding was performed, and themes and subthemes were identified. Thematic analysis used a hybrid approach combining inductive and deductive methods. A constructivist epidemiological approach was opted for this study. In this approach there are five key stages, i.e., generating ideas, exploring concepts, proposing explanations, developing solutions, and taking action. This approach underscores that knowledge attainment is largely an unobtrusive process of learning from the experiences and insights of others, nurturing deeper understanding within the research context (27).

The constructivist worldview is manifest in the phenomenological studies wherein people narrate their experiences. Phenomenology delves into how individuals, perceive, experience, conceptualize, and understand a specific phenomenon (28). This method helps research scholars in apprehending participants particular experiences, which makes it possible to interpret explicit practices, programs, and events in order to acquire in depth understanding on how people construct meaning from their lived experiences within a social context, thereby exploring their inner world. The phenomenological approach employed in this study is interpretive phenomenology, which integrates the researcher’s professional knowledge as an insightful guide to making the analysis eloquent (28).

### 2.1 Ethical considerations

The ethical approval was obtained from The Islamia University Bahawalpur (Reference number: 339/ORIC). All the participants were provided with the participant information sheet, and informed consent was obtained before their inclusion. All the quotes are anonymized as participants 1–10 to protect their identities. In the transcripts, pseudonyms were used, which were stored in the password-protected file. All the data will be kept for 3 years after the study’s completion.

## 3 Results

All 10 participants in this study were professionals with diverse experience in hepatitis work, spanning from 3 to 7 years, with an average experience of 5 years. Six participants were male, and 4 were female (Table 1). Three participants hold a PhD degree in Public Health, and all others had a Master’s degree in Public Health with a basic degree in either MBBS, pharmacy Nursing or any other health-related field.

**Table 1:** Participant information based on gender, stakeholder type and years of experience in hepatitis related work.

Participants	Gender	Years of experience	Type of stakeholder
1	Male	6	Policy maker
2	Male	4	Clinician at hepatitis clinic
3	Male	6	Provincial hepatitis program
4	Female	3	Policy maker
5	Male	3	Provincial hepatitis program
6	Male	7	Provincial hepatitis program
7	Female	5	Provincial hepatitis program
8	Female	6	Policy maker
9	Male	5	Provincial hepatitis program
10	Female	5	Clinician at hepatitis clinic

### 3.1 Perceptions of hepatitis and its elimination program

**Figure 1:**
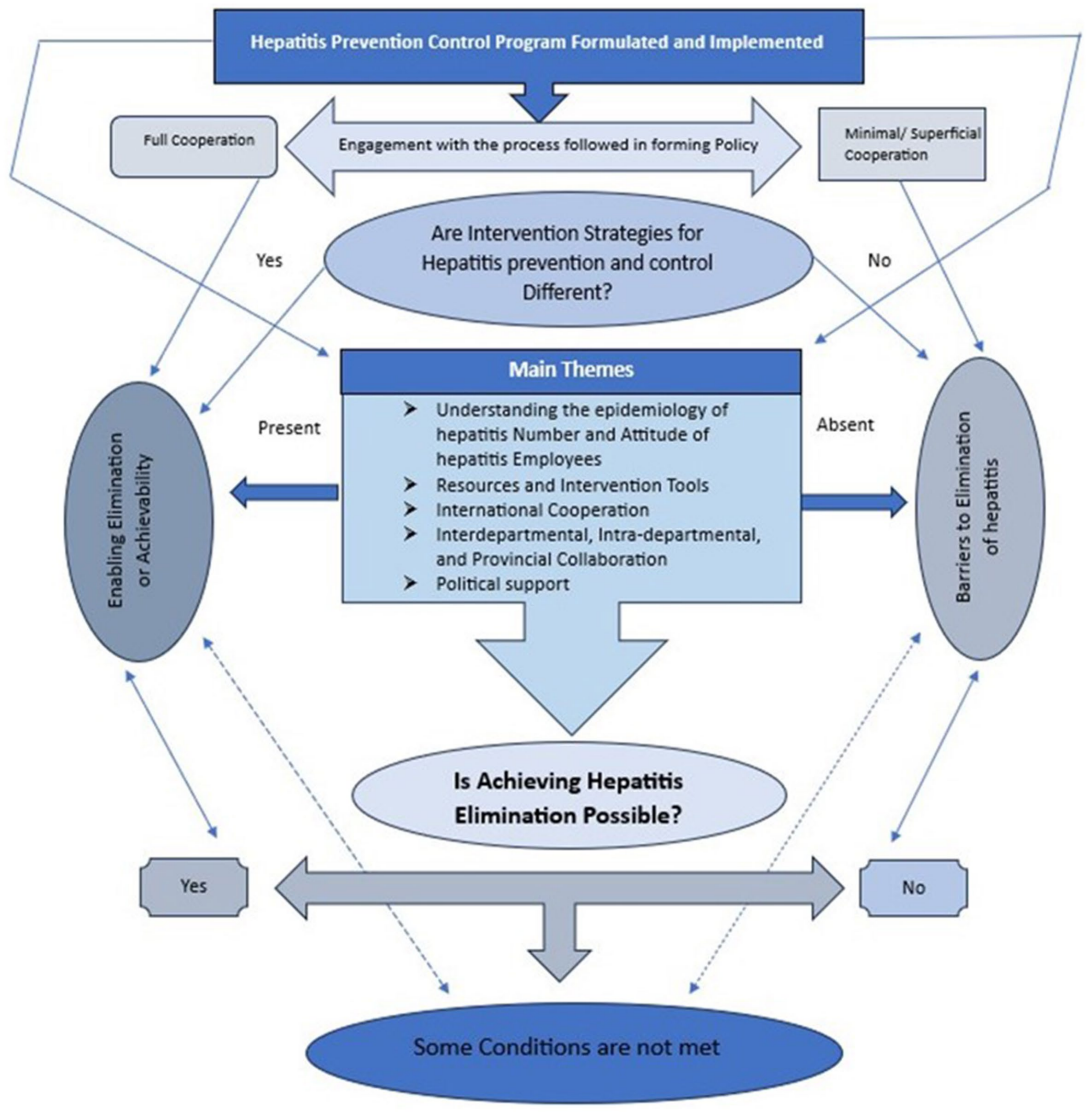
Framework demonstrating features affecting the implementation of hepatitis elimination in Pakistan.

Participants 1–2, 6–9 shared a predominant perspective that hepatitis continues to pose a significant challenge in Pakistan (Figure 1). They expressed concerns over the recent surge in hepatitis cases and related fatalities (participants 3, 5–7, 10). Two of the participants in this study have rejected the notion that any significant progress has been made since the adoption of the hepatitis prevention and control policy in Pakistan (participants 1 and 5).

“We have seen an increase in the cases of hepatitis, which is concerning. Currently, the prevalence of hepatitis C in Baluchistan is 25.7%, Punjab 5.4%, Sindh 2.6% and KPK 6.1%. This is something to worry about, as we had set a target to control hepatitis by 2030” (participant 1).

“Despite the adoption of hepatitis control policies, not much has changed” (participant 5).

### 3.2 Transitioning from hepatitis control and elimination efforts in Pakistan

There were prevalent sentiments indicating that hepatitis intervention strategies and resources in Pakistan have not transitioned adequately from control to elimination efforts (participants 1, 3, 6, 7, 9, 10). “*With the current progress, hepatitis elimination seems impossible*” (participant 4).

“The government has announced the hepatitis elimination program with the ambitious goal to screen almost 135 million people by 2030. Despite this, we are still targeting the same population for testing and treatment as we were six years ago. Even in health promotion, we still use the same messages for hepatitis control and elimination. There are no new messages for hepatitis elimination” (participant 3).

“I have read the policy, and as I say, it annoyed me so much because advocacy strategy and proper action plan are missing. I could not see that it is an elimination policy because it just repeats exactly what we have been doing for the last decade” (participant 8).

### 3.3 The achievability of hepatitis elimination

Three distinct perspectives were found regarding the feasibility of the current hepatitis elimination program, with a general view that 2030 may be too ambitious to achieve a target (participants 1, 3, 10). One viewpoint holds that hepatitis elimination by 2030 is feasible although concerns were raised about the timeline (participants 4). Another group expressed pessimism, doubting the possibility of hepatitis elimination in Pakistan (participant 6). While, the last group believed that hepatitis elimination depends on adequately deploying effective and new intervention measures and resources (participant 1, 8, 9). Others in this study attributed the issue to the stakeholders who focused on controlling hepatitis instead of elimination (participant 2–3, 7). These statements highlight that although hepatitis case incidence in Pakistan meets the WHO threshold for targeting hepatitis elimination, there has been a lack of implementation of appropriate intervention strategies tailored to the requirements of hepatitis elimination.

“I am concerned about the 2030 goals being unrealistic and disappointing. Without funding and government will, we will not be able to achieve the goal. We need to set a clear deadline for hepatitis elimination … Recently, Egypt has set an example by controlling hepatitis to 80%, and it's no different from Pakistan. We lack commitment and will to eliminate hepatitis” (participant 3).

“We are not on the track to eliminate hepatitis till 2030, will not meet the hepatitis elimination targets even if we are given additional thirty years to do so” (participant 6).

“The electronic data collection is present but does not properly monitor the progress of treating and testing hepatitis cases uniformly across the country. Besides this, there is a great shortage of skilled staff. Currently, all the experienced staff members working in the Hepatitis Control Program in Punjab have left, and the department is only run by a few untrained and inexperienced people. How can they run the program? So that is why I am saying, before we talk about hepatitis elimination, we should first ensure that we have skilled staff and management who know their goal and means to achieve it” (participant 1).

Another issue that affects the achievability of hepatitis elimination is collaboration among government entities, provinces, non-government organizations (NGOs) and healthcare companies. Some participants argued that lack of coordination among different provinces poses a significant obstacle to effectively eliminating hepatitis.

“Implementing hepatitis has challenges in that elimination doesn't fully rely on us as workers for the hepatitis control program. It relies on the proper reporting of hepatitis cases. The national database or registry is unavailable, preventing the comprehensive data analysis, tracking the prevalence of hepatitis and high-risk areas identification for targeted interventions” (participant 2).

The significance of international collaboration emerged as a significant influencer on the implementation of hepatitis elimination policy in Pakistan, shaping its success prospects (participants 1–3, 7).

“Pakistan could learn from the Egypt model of combating and eliminating hepatitis by improving the antiviral local production, negotiating the low prices of test kits and collaborating with private, public and nonprofit organizations” (participant 1).

### 3.4 Development process of Pakistan’s hepatitis elimination policy

The view was that hepatitis cases (participants 2, 8), the WHO manual (participants 3, 8, 9, 10), international organizations (participant 4) and the local stakeholders (participants 7–9) were the main drivers in the formation of hepatitis elimination policy.

“Policy development is a highly complex process, as progress toward hepatitis elimination was not merely an idea within the program but also engaged with the WHO and researchers, private sector, experts in hepatitis case management and health promotion, policymakers in the department and the inter-departmental coordinators” (participant 3).

“I think a main error was that the health department put together this agenda of elimination without involving people present on the ground, so they do not know where it is coming from, and they did not have any input” (participant 5).

“We attend many meetings and feel frustrated because they are just swept off the table when we raise critical issues. I personally spent days in modifying the policy drafts, but after four months, the original draft came again and I never saw my input appearing here” (participant 10).

### 3.5 Hepatitis epidemiology in Pakistan

Scrutiny was directed toward the gaps in hepatitis epidemiology in Pakistan, with particular emphasis placed on the major gaps including deficiencies within the surveillance system (participant 4, 10), lack of a national registry (participant 1), inconsistent data and coordination among provinces (participants 1, 5) and lack of comprehensive national strategies (participants 3, 5–7).

“There is a lack of comprehensive studies on hepatitis nationwide. Most data on the prevalence of hepatitis mainly comes from localized studies, small sample sizes and hospital-based data instead of large-scale community-based surveys. This makes it impossible to get an accurate picture of the hepatitis prevalence across the country” (participant 8).

“The absence of a national database registry makes it very hard to comprehensively analyze the prevalence of hepatitis, to identify the high-risk areas and to track the disease. There is also no coordination between different provinces; I am working here in the Punjab hepatitis control program but rarely coordinate with the staff of Sindh or Baluchistan hepatitis control program, which is a great obstacle in the effective management of hepatitis and leads to its underreporting of both cases and mortality rate” (participant 6).

### 3.6 Political dynamics in hepatitis elimination efforts

Three perspectives on the political dimensions of hepatitis elimination emerged, including (i) lack of political will to eradicate hepatitis (participants 3, 5, 8, 9); (ii) inadequate funding and its misuse (participants 2, 3, 9); (iii) Fragmented Healthcare system (Participant 1–3, 7); (iv) development and execution of hepatitis elimination policy in response to global political influence (participant 5); (v) insufficiency of comprehensive policies (participant 8–10).

“While the government has announced the ambitious plan related to hepatitis elimination, the funds are insufficient to yield the expected outcomes. The allocated funds are used for personal benefits. You can say that 40% of the funds allocated for hepatitis elimination are used for personal gain. The government negligence is prominent, as it fails to handle this massive corruption. A proper accountability system is needed urgently to prevent the funds misuse that are intended for hepatitis elimination” (participant 3).

“The government has no concern about the misuse of funds. The national hepatitis framework strategy (2017-2021) has not been updated, to the best of my knowledge. This clearly shows a lack of political commitment in eliminating hepatitis” (participant 2).

“According to the hepatitis elimination strategy 2017–2021, by 2023, almost 70% of the population (140 million) should be tested and treated. However, this goal has not been achieved due to the lack of funds and corruption” (participant 9).

“I never hear anyone from the political side talking about hepatitis elimination because, by now, we should be running awareness campaigns on TV and radio. Minister of Health should take this issue seriously, and electronic campaign should be launched on different media regarding its elimination” (participant 5).

“I think there is some big political agenda behind it. I think it was, to some extent, politically pressurized to form a hepatitis elimination policy for the country. This policy is going to cost so much, but it is not sure that this will work” (participant 9).

“Fragmented healthcare system is another reason for the limited access to hepatitis diagnosis and treatment. Although 281 hepatitis clinics have been established at different DHQs/THQs and teaching hospitals, services are hindered in rural areas due to the fragmented healthcare system” (participant 7).

### 3.7 Key lessons for Pakistan in pursuing hepatitis elimination

Participants highlighted key lessons for Pakistan’s efforts toward hepatitis elimination, including:

- Conduct National Assessment: Gain a thorough understanding of the current hepatitis situation through comprehensive nationwide studies.
- Reduce Dependency on Donors: Avoid dependency on donors for funding and technical inputs and use indigenous expertise and resources to combat viral Hepatitis C.
- Enhance Awareness Campaigns: Emphasize the comprehensive awareness campaigns related to hepatitis while ensuring the involvement of all stakeholders in the elimination agenda.
- Set Realistic Goals: Set cost-effective and realistic goals drawing from the experiences of successful implementation of hepatitis elimination programs in comparable settings.
- Strengthen Surveillance System: Improve surveillance to ensure the early reporting and investigation of the cases.

## 4 Discussion

In concurrence with the findings of previous studies, this research affirms that the feasibility of hepatitis elimination in Pakistan remains a subject of intense discussion. Over the years, hepatitis mortality and morbidity have increased in Pakistan-one in every twenty people has been suffering from HCV infection (14). Given this burden, the elimination of hepatitis C should be a priority. However, evidence shows that eliminating hepatitis by 2030 is highly unlikely. It is important for Pakistan to substantially increase the diagnosis rates and access to treatment to meet the WHO target.

The results of this study uncovered that resource constraints, ineffective intervention strategies, limited staff capacity, corruption, and political apathy jeopardize Pakistan’s ambition to eliminate hepatitis by 2030. Similar observations have been made in other studies, which concluded that lack of political will and resources are major factors in achieving hepatitis elimination (22, 29).

This study’s lack of progress has been attributed to the failure of the policy. The opinion of stakeholders regarding the policy was linked with their perception of the process that was followed in the policy-making. Except for policymakers, other participants perceived that the policy formation process was superficially participatory. All participants from the hepatitis program, however, felt that the meetings conducted were genuinely participatory. Some participants believed that the primary drivers of the policy development process were high-level international organizations such as WHO and Pakistan, which, to some degree, were politically compelled to shape the policy.

The findings of the study have shown that in Pakistan, hepatitis elimination has so far been low on political priority. Owing to poor implementation of government-sponsored treatment programs and health-related policies by devolved provincial health ministries. The findings suggest the urgent need for a central decision-making body to set recommendations and guidelines to simplify the treatment pathway and facilitate mass screening and treatment efforts. Motivation for such a paradigm shift can be taken from Egypt, which have successfully transitioned from having one of the highest burdens of HCV in the world to the lowest by reducing the HCV prevalence from 10 to 0.38% in just 10 years (30, 31).

The study showed that epidemiological data availability, the national policy plan for Hepatitis, awareness of HCV, screening program, guideline on treatment and prevention programs are some of the factors that influence the effectiveness of hepatitis elimination program. Moreover, the national hepatitis registry is also one of the vital components in the program for eliminating viral hepatitis C. Among the various factors that have been identified to have caused the rise in the number of hepatitis C infection cases is the absence of a national database system/data repository in the country. Several countries worldwide are updating the prevalence and program effectiveness through these registries/real time data repositories which depicts true picture of situation in a particular point in time (31, 32).

Presently, owing to devolution of health system in Pakistan since 2010 onwards each province its own program which is entirely running independently of other provinces and the national ministry of health services regulations and coordination, which results in lack of uniform standardized guidelines and policies for screening, testing and management of hepatitis. Keeping in view these facts and the aim hepatitis elimination in Pakistan, it is imperative to take prompt action to establish the national database registry. This intervention will enable assimilation of real time data from all provinces so as to monitor and analyze the rates of hepatitis and identifying the high-risk regions/hotspots. It will also help in prompt resource mobilization and taking immediate measures which will ultimately aid in eradication of hepatitis C. Since 2008, no national level study has been carried out to determine the precise prevalence of hepatitis in Pakistan (15).

The elimination program is premised on fragmented provincial data, which is mainly a passive surveillance system with several gaps and loopholes. Thus, Pakistan requires national hepatitis prevalence data through active surveillance. The creation of a national data-based registry will facilitate cooperation with all provinces, NGOs, and the federal government. For the goal of eliminating hepatitis, there must be a centralized approach that can reduce the disparities in the case definition. Some of the issues that have been highlighted in this study is that unrealistic targets have been set without considering the current epidemiological situation of hepatitis and resources available. Lack of reliable data on hepatitis prevalence and the low-quality system of surveillance limit Pakistan’s capacity to implement, guide and monitor effective responses to hepatitis policy.

To advocate for a proper allocation of resources and mobilize external funding, Pakistan should update the hepatitis strategic framework (2017–2021) and set achievable targets to advance efforts toward hepatitis elimination. A fragmented system of healthcare with a lack of awareness and insufficient training are the main obstacles behind unsuccessful hepatitis elimination programs. For this program to work, it is important to manage the fragmented healthcare system by strong collaborative efforts among the provinces. Elimination of hepatitis needs seriousness and prevention awareness at a grassroot level, and awareness must be created among people about the mode of transmission and preventive measures.

Successful control of hepatitis will depend on strengthening hepatitis control programs, broader access to rapid diagnosis and effective treatment provision. Therefore, fund providers and political commitment are important to prevent the spread of hepatitis. There is a great need for systematic monitoring of the ongoing hepatitis treatment to shed light on the true scale of the issue and to monitor the progress of measures currently being used to address this problem. In addition, Pakistan will need to significantly enhance the efficiency of its healthcare delivery system. The foundation of the elimination hepatitis strategy comprises strong policies, innovation and research, and integrated patient-centric care and prevention, which involve a broad range of stakeholders across the private sector, NGOs and government. Focused efforts to strengthen the primary healthcare system through careful planning, effective implementation and adequate funding can increase the program’s effectiveness.

## 5 Strengths and limitations

The main strength of this study lies in its ability to engage with and analyse the perspectives of key policy-level stakeholders committed to hepatitis C elimination in Pakistan, thereby revealing both convergent and divergent opinions on the barriers and facilitators to policy implementation. The study participants were highly informed about the ongoing policy and response of the government as they were at the helm of implementation. This targeted approach enhances the applicability of the study to policy-level planning and decision-making.

Nevertheless, the study had certain limitations. The purposive sampling of ten informants, while appropriate for the study’s focused objective, represents a relatively small sample and may not capture the full diversity of views among policy actors. Additionally, the research process followed a largely linear structure—completing all interviews prior to analysis—rather than an iterative model, which limited the opportunity to refine data collection based on emerging themes. Despite these constraints, the study provides meaningful findings that can support future policy refinement and implementation strategies.

## 6 Conclusion

The burden of hepatitis C virus in Pakistan is one the highest in the world, with over 8 million people living with HCV infection. The study has provided some key insight into the perspectives of various stakeholders on Pakistan’s hepatitis elimination policy. Despite the adoption of a national elimination strategy, majority of participants expressed skepticism about the feasibility of achieving hepatitis C elimination in Pakistan by 2030. There are host of barriers hindering the achievement of this feat, including insufficient resources, deep rooted governance issues, rampant corruption, lack of political ownership, and a fragmented healthcare system. Pakistan’s mounting hepatitis C burden requires urgent policy action to meet the WHO 2030 elimination goals. Major recommendations include strengthening healthcare governance structure by improving coordination across provinces and establishing a centralized national database for better data management and evidence-based decision making. Policies should entail rapidly expanding diagnostic capacity and ensuring equitable access to treatment nationwide. Strong political commitment and sustained financial support are imperative to address resource and administrative challenges. Additionally, adopting integrated, patient-centered care models and promoting collaboration between government, private sector, and NGOs will be critical to the success of Pakistan’s hepatitis elimination strategy. By bridging these barriers and learning key lessons from other countries, Pakistan can make significant strides toward achieving its hepatitis elimination goals.

## Data Availability

The raw data supporting the conclusions of this article will be made available by the authors, without undue reservation.
